# Implications of endoplasmic reticulum stress and autophagy in aging and cardiovascular diseases

**DOI:** 10.3389/fphar.2024.1413853

**Published:** 2024-07-25

**Authors:** Chenguang Ma, Yang Liu, Zhiling Fu

**Affiliations:** ^1^ Department of Anesthesiology, Shengjing Hospital of China Medical University, Shenyang, China; ^2^ 32295 Troops of P.L.A, Liaoyang, China

**Keywords:** ER stress, UPR, autophagy, aging, cardiovascular diseases

## Abstract

The average lifespan of humans has been increasing, resulting in a rapidly rising percentage of older individuals and high morbidity of aging-associated diseases, especially cardiovascular diseases (CVDs). Diverse intracellular and extracellular factors that interrupt homeostatic functions in the endoplasmic reticulum (ER) induce ER stress. Cells employ a dynamic signaling pathway of unfolded protein response (UPR) to buffer ER stress. Recent studies have demonstrated that ER stress triggers various cellular processes associated with aging and many aging-associated diseases, including CVDs. Autophagy is a conserved process involving lysosomal degradation and recycling of cytoplasmic components, proteins, organelles, and pathogens that invade the cytoplasm. Autophagy is vital for combating the adverse influence of aging on the heart. The present report summarizes recent studies on the mechanism of ER stress and autophagy and their overlap in aging and on CVD pathogenesis in the context of aging. It also discusses possible therapeutic interventions targeting ER stress and autophagy that might delay aging and prevent or treat CVDs.

## Graphical Abstract

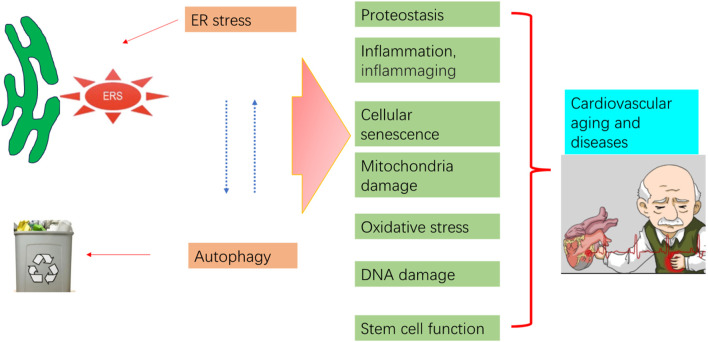

## 1 Introduction

The average human lifespan has been increasing, resulting in a rapidly rising percentage of older individuals and high morbidity of aging-associated diseases, especially cardiovascular diseases (CVDs) (Alfaras et al., 2016). Within this aging population, CVDs are responsible for 40% of all deaths and are the primary reason for morbidity and mortality worldwide. The cost associated with CVD treatment continues to increase (Prince et al., 2015; Gaur et al., 2024). Thus, exploring molecular cues underlying the etiology of aging and its relationship with CVDs has become a public health priority (Marian et al., 2018). Current interventions mainly focus on managing and preventing comorbidity while neglecting the nature of the aging process (Alfaras et al., 2016).

The endoplasmic reticulum (ER) is a network of branching tubules and flattened sacs in eukaryotic cells governing protein synthesis, Ca^2+^ storage, lipid formation, and signaling (Hetz et al., 2020). Diverse intracellular and extracellular factors interrupting any homeostatic functions in the ER can induce ER stress. To mitigate ER stress and regulate the protein-folding ability of the ER to alter secretory demands, the cells adopt a dynamic signaling pathway of unfolded protein response (UPR) (Hwang and Qi, 2018). However, persistent UPR can cause a maladaptive response (Wiseman et al., 2022). Chronic and sustained ER stress and proteostasis defects are representative features of numerous diseases, including inflammatory diseases, metabolic diseases, cancers, and age-associated diseases like CVDs (Wang and Kaufman, 2012; Uddin et al., 2021).

Autophagy is a highly conserved process that involves lysosomal degradation and recycling of cytoplasmic components, proteins, organelles, and pathogens that invade the cytoplasm (Levine and Klionsky, 2017). Autophagy’s function in maintaining proteostasis, together with the observation that autophagy declines with aging, links it to proteostasis loss, which is a typical characteristic of aging (Kern and Behl, 2019). In addition, this core molecular pathway is critical for orchestrating cellular responses to stress, cellular reprograming, maintaining stemness, fine-tuning senescence, and modulating inflammation (Kaushik et al., 2021). Changes in all of these processes accompany the aging process. Active autophagic activities have been discovered in all cardiovascular tissues (Lavandero et al., 2015). It is also possible that autophagy is vital for combating adverse influences of aging on the heart, while autophagy and autophagic flux generally decrease in aging hearts (Shirakabe et al., 2016).

It is now apparent that ER stress can be a potent trigger for autophagy (Rashid et al., 2015; Jiao et al., 2023), and the interaction of ER stress and autophagy may be implicated in the etiology of aging and CVDs. Therefore, methods of modulating ER stress/UPR, autophagy, and their interaction may serve as therapeutic targets of clinical relevance in aging and aging-related CVDs.

The present report summarizes recent studies on the mechanism of ER stress and autophagy and their overlap in aging and on CVD pathogenesis in the context of aging. Possible therapeutic interventions targeting ER stress and autophagy that could be employed to delay aging and prevent or treat CVDs are also discussed.

The cardiovascular system functions by delivering oxygenated blood to the body’s tissues. Maintaining homeostasis is critical for tissue health and organism longevity. With age, the heart undergoes complicated changes that influence cellular function, ultimately transitioning from a compensatory adaptive to a decompensatory maladaptive state. Both the absolute number of cardiomyocytes and their repopulation from cardiac stem cell reserves decline due to an increase in apoptosis and necrosis (Goldspink et al., 2003), leading to a decrease in myocardial contractile capacity, increase in left ventricular wall thickness, and elevation in chamber dimension, prolonging diastole and ultimately damaging the cardiac pump function (Obas and Vasan, 2018). Therefore, aging cardiomyocytes become more susceptible to stress. Aging is also associated with vascular structure changes and declined function, particularly in the great arteries (Safar, 2010; Kovacic et al., 2011). Aging-related impairment of vascular structural changes and functional decline (van der Linden et al., 2024) is a cause of morphological changes that include vascular wall thickening, perivascular fibrosis, collagen deposition, and vessel dilatation (Costantino et al., 2016). Thickening is a critical factor in aged vasculature that promotes arterial stiffness, which, in turn, increases pulsatile and left ventricular systolic loads (Boutouyrie et al., 2021).

## 2 ER stress, UPR, autophagy, and aging

### 2.1 Overview of ER stress and UPR

ER is a double-membrane bound organelle promoting numerous functions, such as protein folding, Ca^2+^ homeostasis, and lipid/sterol synthesis. Several chaperones, foldases, and cofactors play a central role in carrying out these functions in the ER. Pathological and physiological insults, including oxidative stress, perturbation in Ca^2+^ homeostasis, release of inflammatory cytokines and toxins, and aging-related insults, can cause a buildup of unfolded and misfolded proteins in the ER. This buildup then leads to downstream disruptions in the integrity and function of the secreted proteome, a condition known as ER stress.

PKR-like ER kinase (PERK) activation through autophosphorylation and homodimerization leads to signaling cascades that include direct phosphorylation downstream of eIF2α, triggering a total downregulation of translation and inhibition of protein synthesis. The eIF2α phosphorylation augments ATF4 translation. ATF4 triggers the CCAAT enhancer-binding protein (C/EBP) homologous protein (CHOP), which translocates to the nucleus. Bcl-2 protein family then induces apoptosis.

Binding immunoglobulin protein (BiP) dissociation triggers IRE1 oligomerization and trans-autophosphorylation, activating endoribonuclease activities and promoting XBP1 mRNA splicing. Spliced XBP1 (sXBP1) is a crucial transcription factor that induces gene expression of ER chaperones and multiple UPR target genes, thereby boosting the ER folding ability.

BiP dissociates from ATF6α, exposing its Golgi localization signal and resulting in the translocation of ATF6α from the ER to the Golgi apparatus, where it is sequentially cleaved by site-1 S1P and S2P, liberating the cytoplasmic ATF6α segment. The activated ATF6α fragment later binds to cis-acting ER stress response elements in the nucleus, increasing genetic expression of UPR proteins, including BiP, CHOP, GRP94, and ER-associated degradation (ERAD) components, suppressing ER stress, and restoring protein-folding homeostasis.

**FIGURE 1:**
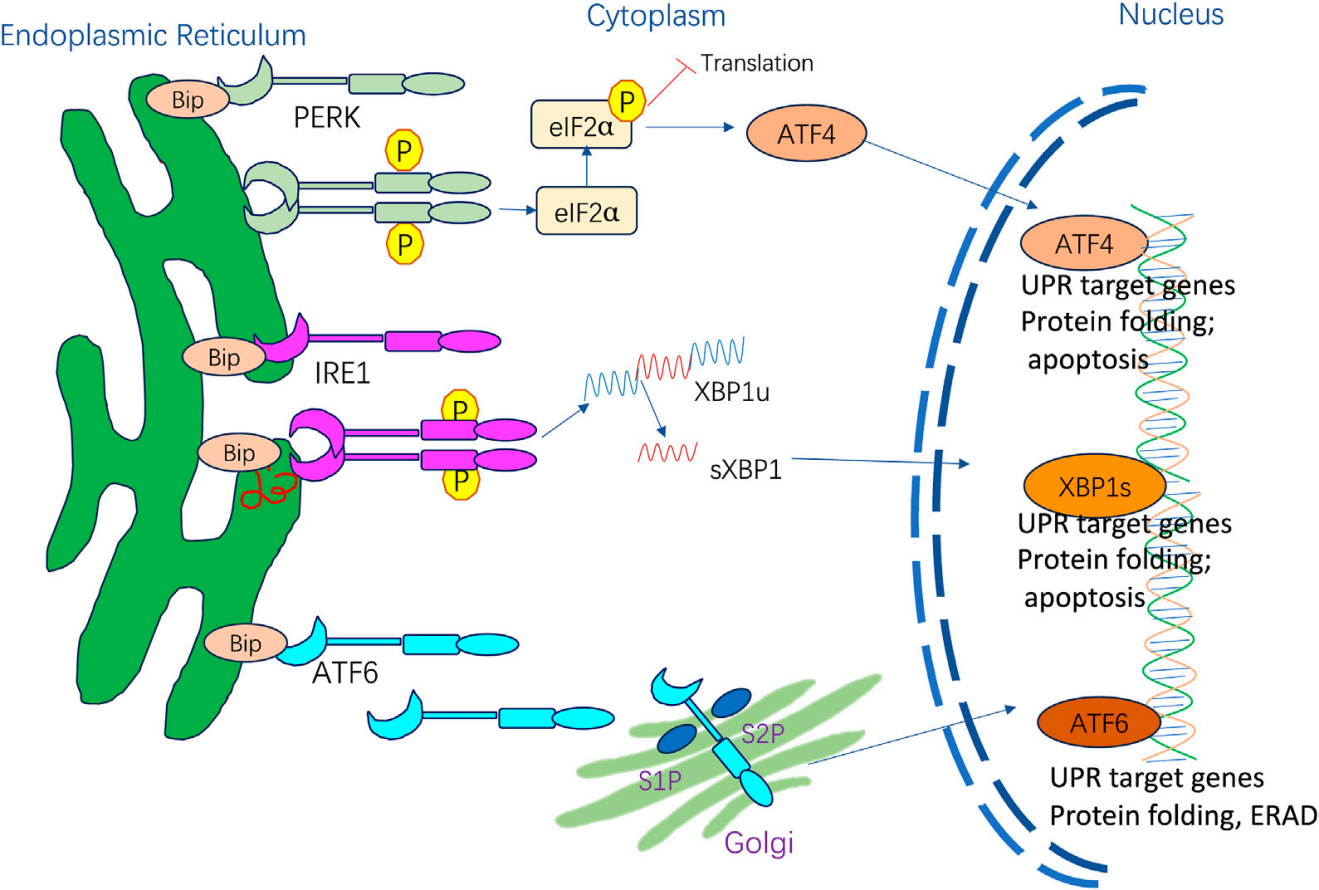
ER stress and activation of UPR signaling pathways.

The adaptive UPR is vital for restoring ER homeostasis after the accumulation of potentially toxic misfolded proteins (Hetz, 2012; Hetz et al., 2020). In mammalian cells, the UPR is regulated by three ER membrane-embedded sensors: PKR-like ER kinase (PERK), inositol-requiring enzyme 1 (IRE1), and activating transcription factor 6 (ATF6) (Figure 1). These proteins can act individually or synergistically to alleviate ER stress on the organism (Spencer and Finnie, 2020). In basal conditions, transmembrane protein activation is restrained by the binding of ER-resident BiP GRP78. When ER stress is present, BiP dissociates and activates PERK, IRE1, and ATF6 (Hetz, 2012). The three mediators directly activate a full transcriptional and translational signaling program, acting on protein secretion, redox homeostasis, lipid biosynthesis, and cell death programs.

PERK is activated via homodimerization and autophosphorylation when BiP is released, resulting in a signaling cascade that includes direct phosphorylation of downstream eukaryotic translation initiation factor 2α (eIF2α) and triggering a full downregulation of translation and protein synthesis inhibition (Hetz et al., 2020). The eIF2α phosphorylation can also increase the translation of specific mRNAs with upstream open reading frames like transcription factor 4 (ATF4) (Vattem and Wek, 2004). ATF4 triggers the induction of one pro-apoptotic transcription factor known as CHOP under sustained stress conditions (Hetz et al., 2020). CHOP translocates to the nucleus and controls the components of the B-cell lymphoma protein 2 (Bcl-2) protein family to induce ER stress-mediated apoptosis. Moreover, phosphorylated eIF2α acts on UPR termination by inducing the growth arrest and DNA damage-inducible protein 34 (GADD34) (Yang et al., 2024).

IRE1 is a type I transmembrane protein with two enzymatic activities: it functions as a serine/threonine kinase and a cytosolic endoribonuclease (Grey et al., 2020). BiP dissociation with IRE1 triggers IRE1 oligomerization and trans-autophosphorylation, resulting in the activation of endoribonuclease activity (Karagöz et al., 2017) and promoting splicing of the 26-base intron from X-box-binding protein 1 (XBP1) mRNA (Urra et al., 2020). sXBP1 is a crucial transcription factor that induces gene expression of ER chaperones and multiple UPR target genes to boost the ER folding ability. sXBP1 is also involved in cell survival, differentiation, and development (Almanza et al., 2019). Moreover, IRE1 cleaves and downregulates microRNAs and mRNAs via its RNase domain and IRE1-dependent decay (RIDD). When ER stress persists, RIDD activity is increased, and XBP1 mRNA splicing is decreased, aggravating uncontrolled cell death (Hollien and Weissman, 2006; Wiese et al., 2022).

ATF6 is a type II transmembrane protein with a basic leucine zipper motif. BiP dissociates from ATF6α upon ER stress, exposing the Golgi localization signal of ATF6α and causing its translocation from the ER to the Golgi apparatus. This facilitates proteolytic removal of luminal and transmembrane domains by site-1 protease (S1P) and site-2 protease (S2P) (Ye et al., 2000), liberating the cytoplasmic ATF6α segment, becoming activated to allow interactions with cis-acting ER stress response elements in the nucleus, and enhancing genetic expression of BiP, GRP94, and CHOP to suppress ER stress (Jin et al., 2017).

In the past 5 years, many studies have reported that UPR components have multiple functions beyond maintaining ER proteostasis. UPR binding with novel partners serves as a platform for interorganelle communication and signaling crosstalk to mediate cytoskeleton dynamics, mitochondrial bioenergetics, and membrane contacts (Carreras-Sureda et al., 2019; Shin et al., 2019; Dufey et al., 2020).

### 2.2 Autophagy overview

Autophagy in mammals can be roughly categorized into three groups based on distinct mechanisms of cargo sequestration. Microautophagy is the process of invagination where cytoplasmic components directly enter the lysosomes, while chaperone-mediated autophagy (CMA) includes selective degradation of target proteins that are recognized and translocated to lysosomes by the chaperone complexes. Macroautophagy (hereafter referred to as autophagy) is another mechanism of adaptation to harsh environmental conditions and intrinsic stress (Mizushima, 2018). Autophagy is a well-defined process mediated by numerous proteins encoded by autophagy-related genes (ATGs).

**FIGURE 2:**
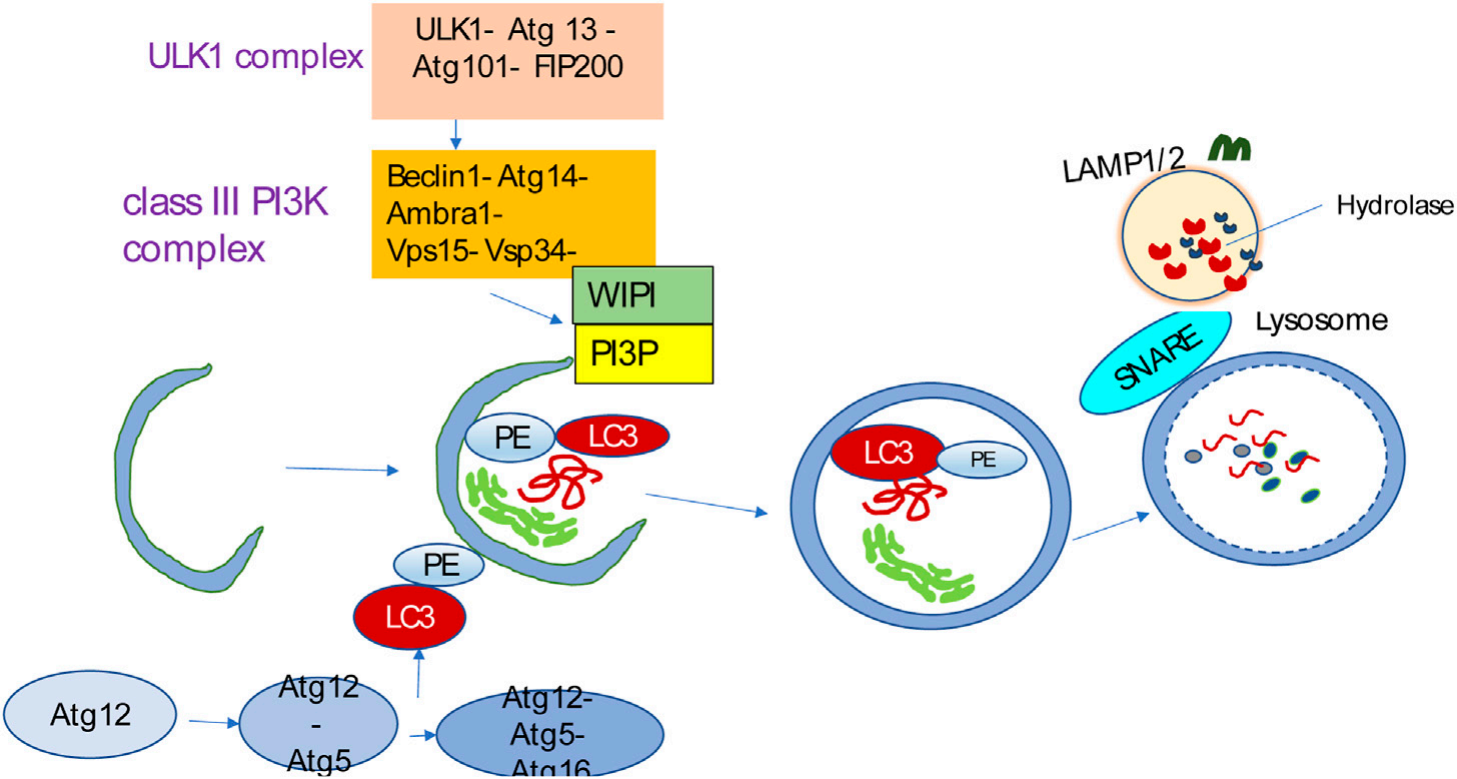
Autophagy overview. Autophagy is initiated by the ULK1 complex and, in turn, activates the class III PI3K complex known as the VPS34 complex, containing VPS34, VPS15, Beclin1, Atg14L, and Ambra1. The VPS34 complex catalyzes the formation of PI3P on the nascent phagophore’s membrane. PI3P facilitates phagophore nucleation and recruits WIPI. WIPI then recruits the ubiquitin-like conjugation systems that promote LC3 conjugation to the phagophore membrane. The LC3-PE conjugation site is marked by the Atg12-Atg5-Atg16L complex that is specific for E3-like ligase to generate LC3-PE. The LC3-PE complex acts as a phagophore receptor and facilitates selective uptake by interacting with adapter molecules. The phagophore matures into the autophagosome, eventually fusing with the lysosome and causing the degradation of sequestered substances by diverse hydrolases. The resulting breakdown products are then released into the cytosol for recycling. SNARE-like proteins promote the fusion of autophagosomes and lysosomes.

The process of autophagy involves initiation, phagophore formation and elongation, and autophagosome–lysosome fusion (Melia et al., 2020) (Figure 2). Membrane assembly and autophagosome formation as a double-membrane phagophore, which completely or partially sequesters cytoplasmic organelles and then fuses to lysosomes, are the two key steps during autophagic flux. Various biological byproducts are released for energy production and recycling via these processes. Autophagy mediates the breakdown of long-lived proteins, in contrast to the turnover of short-lived proteins, which is mediated by other catabolic mechanisms, such as the ubiquitin-proteasome system (UPS) (Gavilán et al., 2024).

Autophagy can be categorized as non‐selective or selective based on the nature of the cargo content. The molecular mechanism of the autophagy process remains basically identical in both types. However, selectivity in selective autophagy is a highly spatiotemporally controlled metabolic pathway guaranteed by specific labeling of every cargo based on stress type and involvement of receptors (or adaptors) (Lamark and Johansen, 2021). Accumulating evidence has shown that abnormalities of selective autophagy are closely related to the occurrence and progression of aging-related diseases, such as cancer, metabolic diseases, neurodegenerative diseases, and CVDs (Ma et al., 2023; Ma et al., 2024).

Autophagy is induced in reaction to diverse stressors, including nutrient or growth factor deprivation, protein and organelle damage, oxidative stress, inflammation, ER stress, and genotoxic stress (Morishita and Mizushima, 2019). Nutrient-sensing pathways, including those involving the mechanistic target of rapamycin complex 1 (mTORC1) and AMP-activated kinase (AMPK), are central to autophagy regulation (Kitada and Koya, 2021). Therefore, identifying the nutrient resources and energy for cells under harsh conditions is important (He, 2022; Feng et al., 2024).

### 2.3 Relationship among UPR, autophagy, and aging

#### 2.3.1 ER stress and UPR during aging

A progressive functional decrease in the ER occurs with age (Bouska et al., 2019). Chaperones, in particular, are progressively oxidized during the aging process, bringing about a functional decline and matching the discovered impairment in adequate cellular reaction to ER stress in cardiac aging (Gude et al., 2018; Li et al., 2019). For example, the function of chaperones like calreticulin, calnexin, and BiP is compromised with age, elevating the number of misfolded or unfolded proteins and protein aggregates. Across the lifetime of Wistar rats, the expression of BiP protein in the brain and peripheral tissues, including lung, kidney, liver, heart, and spleen, was higher in young tissues than in aged tissues (Hussain and Ramaiah, 2007).

Mild and moderate ER stress may induce stress resistance that can extend the lifespan, but prolonged and overwhelming stress may accelerate the aging process (Salminen and Kaarniranta, 2010). On the other hand, aging leads to increased ER stress (Chen et al., 2023). For example, CHOP and caspase-12 can be induced in aged and stressed rats but not in their young and stressed counterparts (Paz Gavilán et al., 2006).

In addition to affecting ER function, aging can influence the ER structure within the cell (Erickson et al., 2006; Moltedo et al., 2019). It has been observed that the highly ordered parallel cisterna of the rough ER, which is a feature of young neurons, becomes dispersed in aged neurons (Hinds and McNelly, 1978). Likewise, an aging and failing heart undergoes architectural changes in the levels of ER and UPR components in cardiomyocytes (Ortega et al., 2014).

#### 2.3.2 Autophagy during aging

Autophagy is crucial for proteostasis maintenance by removing long-lived or damaged proteins and organelles. However, autophagy function goes beyond proteostasis maintenance (Levine and Klionsky, 2017). Autophagic function declines with age, while autophagy dysfunction promotes premature aging. Autophagy inhibition by DBI/ACBP is involved in aging and CVDs (Montégut et al., 2023). Similarly, mice with cardiac-specific deleted ATG5 protein develop dilated cardiomyopathy with serious systolic dysfunction in aging, which accompanies dysfunctional and abnormal mitochondrial accumulation and sarcomeric disarray (Taneike et al., 2010). Mitophagy may also be impaired during cardiac aging. Young Parkin knockout mice exhibited a normal cardiac phenotype, while abnormal mitochondria accumulated in cardiomyocytes with age (Kubli et al., 2013a).

#### 2.3.3 Interrelationship among ER stress, UPR, autophagy, and aging

Autophagy correlates with the ER on numerous levels. These two systems are dynamically connected, and changing the function of one affects the other.

The ER acts as a potential membrane source and scaffold for autophagosome generation. Molecules responsible for the autophagy cascade from ULK1 to PI3P effectors are localized in the ER and contribute to autophagosome nucleation.

PERK has regulatory functions in autophagy mediated by ATF4 and CHOP, which have been shown to transcriptionally adjust in 12 ATG genes. ATF6 upregulates DAPK1 expression, phosphorylating Beclin-1 and mediating autophagy initiation. IRE1 recruits TRAF2 and ASK1, activating c-Jun N-terminal kinase (JNK), which mediates Bcl-XL/Bcl-2 phosphorylation and causes Beclin-1 dissociation and enhanced basal autophagy. sXBP1 specifically binds to the Beclin-1 promoter and induces transcription, causing autophagy upregulation. ER stress can also activate autophagy by promoting the release of a substantial amount of Ca^2+^ from the ER into the cytoplasm.

The ER has been observed in close vicinity to the isolation membrane, serving as a major source of autophagosomes and contributing to autophagosome formation (Senft and Ronai, 2015).

When stress stimuli remain or become extremely strong, UPR and UPS are incapable of restoring the ER to its normal state, and autophagy comes into play, engulfing the damaged ER for degradation. The degraded ER fragments can then be recycled into a fresh ER with restored function. Autophagy becomes the last resort to restore ER homeostasis (Qi and Chen, 2019).

**FIGURE 3:**
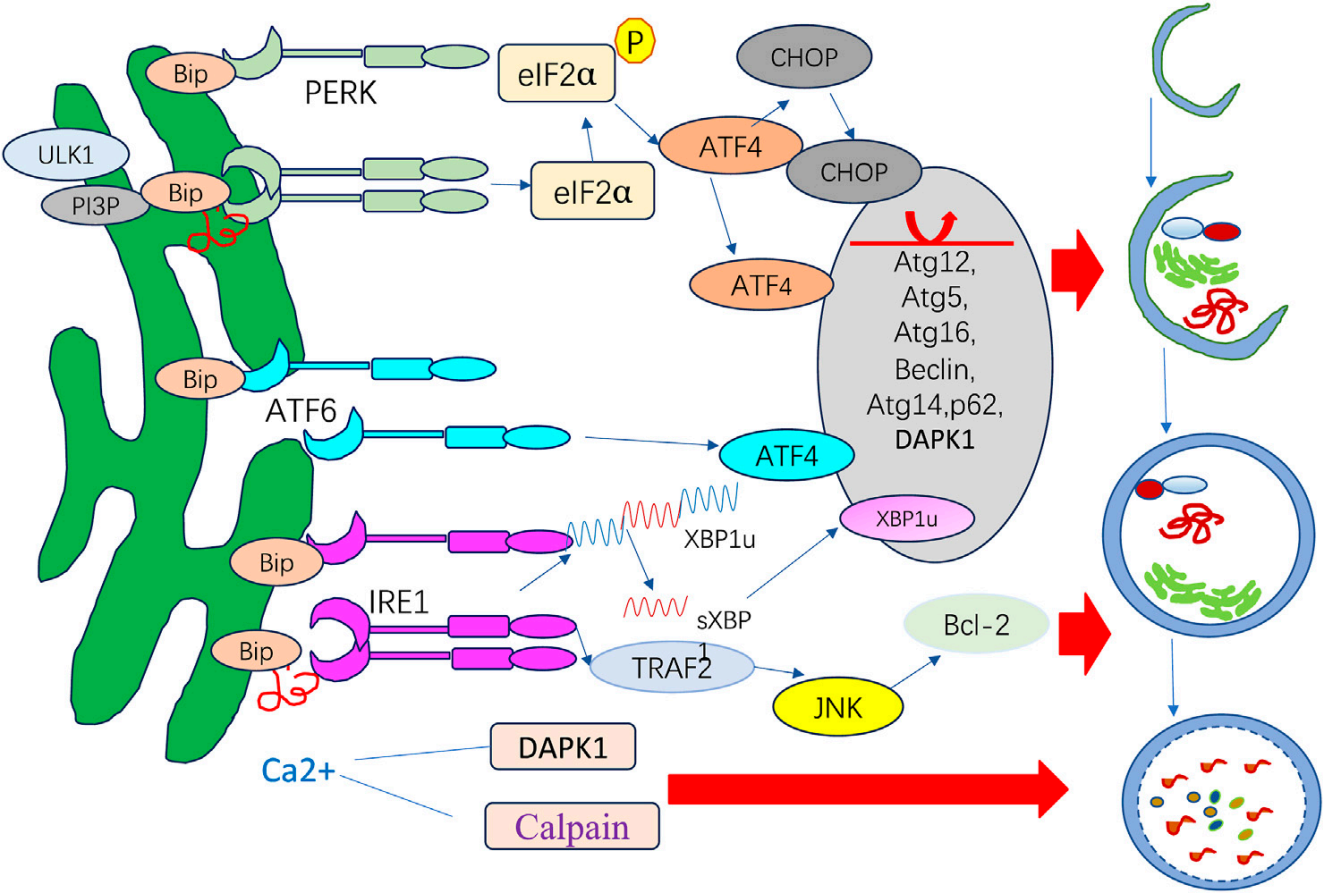
ER stress response and autophagy.

ER stress is a potent trigger for autophagy. In addition to nutrient deprivation and mTOR inhibition, UPR is another factor responsible for autophagy induction (Kapuy, 2024). UPR activation can trigger the change in autophagy that can modulate UPR, exemplifying the crosstalk process. Three UPR arms, including PERK, IRE1α, and ATF6, each facilitate autophagy differently in ER stress. The signaling pathways operate under diverse stress circumstances and in a context-dependent manner (Figure 3).

The eIF2α phosphorylation in worms is critical for the induction of stress granules, which are required for prolongevity using dietary restriction (Kuo et al., 2020). The eIF2α phosphorylation is indispensable for autophagy activation (Kuo et al., 2020). Autophagy is a major anti-aging mechanism, suggesting an interrelationship between UPR and autophagy in pro-survival signals (López-Otín et al., 2023). The activation of mTOR can inhibit autophagy activation, increasing the severity of ER stress. Therefore, there is reciprocal regulation and interaction between ER stress and autophagy (Shi et al., 2024). During the aging process, the ERAD degradation pathway fails to eliminate aggregated misfolded proteins, and the UPR triggers the autophagy process to facilitate their degradation to maintain proteostasis (Pintado et al., 2017). On the other hand, autophagic functions, such as the synthesis rate of autophagosome and the effectiveness of autophagosome fusion with lysosome, are impaired with aging (Kaushik et al., 2021). UPR hyperactivation in the heart induces Sirtuin 1 (SIRT1), which inhibits the ER stress-triggered apoptosis by regulating the PERK activity and deacetylating eIF2α to decrease the proapoptotic protein CHOP expression (Prola et al., 2017; Pires Da Silva et al., 2020). Moreover, SIRT1 can activate autophagy, playing a positive role in various ER-induced damage processes in the aging heart, decreasing apoptosis, regulating UPR sensors, and decreasing the S-nitrosylation effect of protein disulfide isomerase (PDI) (Hsu et al., 2017; Prola et al., 2017). Therefore, ER stress correlates with autophagy via SIRT1 in the heart.

## 3 Mechanism of ER stress, UPR, and autophagy in aging and CVDs

**FIGURE 4:**
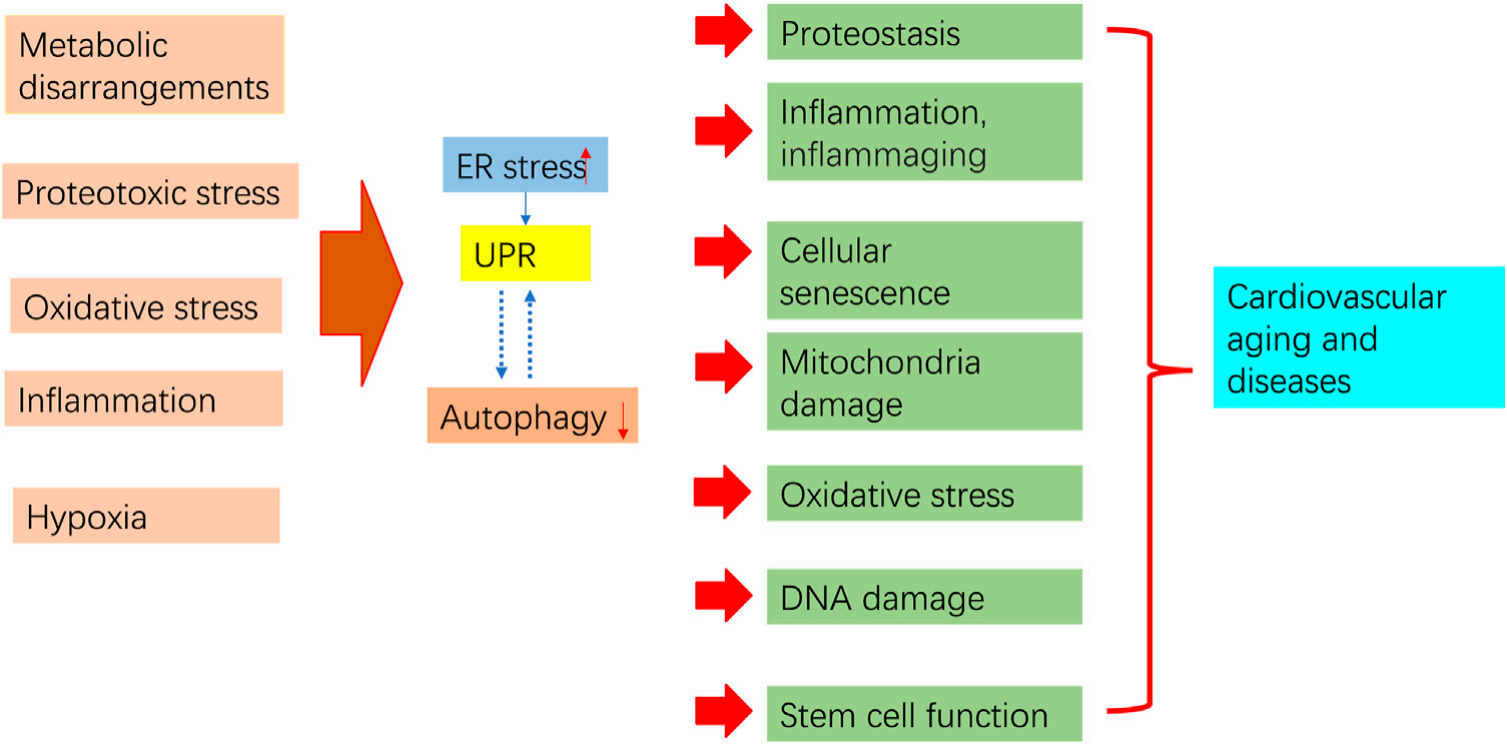
Aging process regulation by ER stress and autophagy. Pro-aging insults impair autophagy and trigger ER stress and subsequent UPR. Prolonged ER stress, impaired autophagy, and their interaction contribute to pathological consequences that may increase aging in the cardiovascular system.

ER stress and autophagy dysregulation are closely interrelated with various cellular stress responses, including inflammation, proteostasis loss, cellular senescence, mitochondrial dysfunction, and oxidative stress (Figure 4).

### 3.1 ER stress, UPR, autophagy, and their overlap in proteostasis

The proteostasis network sustains a normal proteome by integrating protein synthesis, folding, trafficking, and degradation (Hetz and Saxena, 2017). ERAD, UPR, and the autophagy pathway are the main players in this network. Cellular proteostasis derangement has been proposed to be one of the major hallmarks of aging (López-Otín et al., 2013; López-Otín et al., 2023). Healthy proteostasis in cardiomyocytes ensures proper contractile function (Li et al., 2019). Genetic anomalies or environmental factors often causing wear and tear can lead to loss of protein patency and disrupt protein quality control, triggering proteotoxic stress and ultimately resulting in cardiac aging (Henning and Brundel, 2017).

A disruption in ER stress/UPR and autophagy can result in protein quality control failure (Henning and Brundel, 2017; Li et al., 2019). About a third of the proteome is synthesized in the ER, which is vital for folding and modifying secretory and membrane proteins, thereby sustaining proteostasis. ER stress-induced UPR enhances the ER protein-folding capacity by focusing cellular resources to synthesize the proteins involved in the folding process, such as PDIs and ER-resident chaperones (Kilberg et al., 2009). The ER protein-folding load is decreased by inhibiting the whole cellular protein synthesis (Kilberg et al., 2009). Therefore, these aspects of UPR eventually facilitate ER stress relief (Ron and Walter, 2007). However, as with numerous other signaling pathways, UPR is susceptible to aging-associated impairment and becomes less efficient over time (Lim et al., 2023). The aging process is accompanied by an imbalance between augmented ER stress and diminished UPR, leading to chronic ER stress (Brown and Naidoo, 2012) and ultimately causing loss of proteostasis. Therefore, mild cardiac ER stress likely helps to clear damaged, unfolded, or old proteins, thus sustaining appropriate heart functions. However, cardiomyocytes may undergo apoptosis when ER stress is prolonged and persistent. Because cardiomyocytes cannot regenerate, apoptosis harms the heart. Therefore, prolonged ER stress must be avoided (Li et al., 2019).

During the extended period of cellular proteotoxic stress, UPR and UPS become overwhelmed and exhausted. Autophagy is activated to clear misfolded proteins to ensure cell homeostasis and survival (Li et al., 2018a). In all cell types, including cardiomyocytes, autophagy has become a proteasome substitution for the clearance of misfolded proteins from the ER via ERAD by coordinating with other components of the proteostasis network (De Leonibus et al., 2019). In the heart, autophagy mediates the effective clearing of abnormal and damaged proteins and organelles from cardiomyocytes and serves as a cellular recycling mechanism by reclaiming lipids, amino acids, and other molecular building blocks with lysosomal acidic hydrolases, thereby maintaining healthy heart function (Sciarretta et al., 2018). Enhancing autophagy in the aged mouse heart can reduce protein damage, attenuate hypertrophy, and restore Ca^2+^ homeostasis, thus improving contractile function (Taneike et al., 2010). By eliminating defective/damaged mitochondria, mitophagy facilitates mitochondrial proteostasis in response to mitochondrial stress (Pickles et al., 2018), thus conserving normal cardiac contractility and maintaining heart function. Autophagy impairment can disrupt protein degradation and misfolded or damaged protein processing, consequently contributing to aging, pathogenesis, and development of CVDs. Additionally, impaired autophagy also affects the function of UPS due to decreased degradation of damaged or aged proteasomes, severely increasing the levels of proteasome substrates and leading to heart senescence and cardiac disease onset (Korolchuk et al., 2009).

### 3.2 ER stress, UPR, autophagy, and their overlap in inflammation

Chronic inflammation has been identified as a determinant of aging and plays a central role in most aging-associated chronic disorders (Liberale et al., 2022). A recent study has highlighted the function of inflammation as a critical contributor underlying the mechanism conferring cardiovascular risk (Madhur et al., 2021). For instance, it has been determined that inflammation is a major contributor to atherosclerosis from its initiation to progression and, eventually, to its endpoint (Weber et al., 2023). The term “inflammaging” describes chronic, low-grade, systemic inflammation that occurs in older individuals even without overt infections. It is a risk factor for CVDs and a global indicator of poor health status (Ruparelia et al., 2017). Inflammaging is believed to originate from an imbalance between the generation and disposal of damage-associated molecular patterns (DAMPs) (“enemy within”), including cellular debris, misfolded proteins, and/or misplaced self-molecules, and it increases with age (Franceschi et al., 2017). DAMP accumulation can also be sensed by the NLRP3 inflammasome, cause NLRP3 oligomerization, and increase caspase 1-dependent secretion of IL-1β and IL-18. Blockade of the NLRP3 inflammasome prolongs the health span, alleviating multiple aging-related degenerative changes associated with inflammaging (Ferrucci et al., 2005; Youm et al., 2013).

Inflammatory mediators can sustain and even exacerbate ER stress within tissues. Chronic ER stress also facilitates chronic inflammation. Therefore, there may be a positive feedback loop between ER stress and inflammation. Both ER stress and maladaptive UPR can boost inflammation and apoptosis in the heart. Proinflammatory cytokines, including CCL2, IL-8, interferon-γ, and TNF, provoke cytotoxicity via IRE1α and XBP1 (Salminen et al., 2020), while cardiac-specific *Ccl2* overexpression upregulates the expression of ER stress-associated genes and exacerbates IR injury (Zhang W. et al., 2020). ER stress and UPR take part in inflammation via numerous regulators, including nuclear transcription factor-κB (NF-κB) and the NLRP3 inflammasome, and can intervene in Ca^2+^ homeostasis. NF-kB activation mediates myocardial and vascular inflammation in aging-related diseases (Seals et al., 2011). Aging-associated NF-κB signaling upregulates the expression of proinflammatory genes, such as ILs, TNF-α/β, chemokines, and adhesion molecules (Chung et al., 2019). IRE1α may drive NF-κB activation by cooperating with TRAF2 (Shen et al., 2014). CHOP can also stimulate NF-κB signaling in chronic inflammatory diseases (Willy et al., 2015). ER stress can induce inflammation through TXNIP-NLRP3-IL-1β signaling and likely participates in CVD pathogenesis (Yang et al., 2020).

Autophagy is a basic biological process that facilitates innate and adaptive immunity. Autophagy perturbation results in repercussions in diseases with inflammatory components, infections, metabolic disorders, neurodegeneration, autoimmunity, cancer, and CVDs (Deretic, 2021). Autophagy controls the burden imposed by exogenous hazards and infection, as well as by endogenous sources of inflammation, such as molecular aggregates and damaged organelles, while simultaneously limiting the inflammatory process (Matsuzawa-Ishimoto et al., 2018). Autophagy facilitates the recycling of cellular content, maintains homeostasis, and prevents recognition of PAMPs by PRRs and consequent inflammation (Zitvogel et al., 2010). Therefore, autophagy directly inhibits the development of the inflammaging process. Specifically, mitochondria are involved in the proper signaling of some innate immunity platforms like mitochondrial antiviral signaling. They can also be the sources of DAMPs under pathological conditions (Youle, 2019). Damaged mitochondria trigger the NLRP3 inflammasome. By effectively sequestering damaged mitochondria leaking DNA and reactive oxygen species (ROS), mitophagy removes the key signal that promotes NLRP3 inflammasome activation (Matsuzawa-Ishimoto et al., 2018).

The autophagic response diminishes with age, and this impairment may result in inflammasome activation and chronic inflammatory responses, accelerate the aging process, and affect diseases with inflammatory components, including CVDs (Deretic, 2021). CMA function becomes impaired during atherosclerosis progression, leading to increases in NLRP3 inflammasome activation and IL-1β secretion, boosting vascular inflammation and atherosclerosis progression (Qiao et al., 2021).

Both ER stress and autophagy are implicated in inflammation and may be interconnected with pathophysiological aspects of the cardiovascular system. For example, recent research has shown that inhibition of transcription factor CCAAT/enhancer-binding protein beta prevents atherosclerosis development and foam cell formation. Its protective role in atherogenesis involves reducing inflammation, ER stress, and apoptosis, promoting autophagy, and inactivating mTOR (Zahid et al., 2020). Similarly, the protective effect of cinnamaldehyde in CVD progression results in the inhibition of inflammation and oxidative stress and modulation of autophagy and ER stress (Lu L. et al., 2022). However, it is unclear whether autophagy is induced by elevated ER stress or is simply a reaction to injury in these pathological conditions. Nonetheless, both the UPR pathway and autophagy are vital for regulating the inflammation process and are implicated in the aging process and pathogenesis of various diseases, including CVDs.

### 3.3 ER stress, autophagy, and their overlap in cellular senescence

Cellular senescence features include irreversible cell-cycle arrest, apoptosis resistance, tumor suppressor pathway activation, altered metabolism and cell shape, and secretion of numerous proinflammatory cytokines, chemokines, and growth factors, collectively named the senescence-associated secretory phenotype (SASP) (Kuilman et al., 2010). Senescence was first identified in human diploid fibroblasts that showed a finite ability for cell division due to telomere shortening (replicative senescence) (Hayflick and Moorhead, 1961). Senescence independent of telomere length was later discovered within numerous aged or damaged tissues and was critically linked with cellular stresses (Kuilman et al., 2010).

Evidence has shown that cellular senescence exists in a variety of cell forms in the cardiovascular system and accumulates in culprit lesions, playing critical roles in CVD onset and outcome (Wang J. et al., 2015; Song et al., 2020). For example, cells in advanced atherosclerotic plaques usually display markers of senescence, including p16^INK4A^ and tumor suppressor ARF, and express SASP, which further fuels inflammation and produces metalloproteinases that degrade the extracellular matrix, destabilizing atherosclerotic plaque (Wang M. et al., 2015; Yan et al., 2021).

Cellular senescence is closely related to ER stress and UPR. Various cell types undergoing senescence show structural changes in the ER and elicit UPR activation (Abbadie and Pluquet, 2020). The loss of proteostasis is present in senescent cells, which can be attributed to changes in UPR (Sabath et al., 2020).

UPR activation likely occurs in all forms of senescence under various stresses (Benvenuti et al., 2002; Dörr et al., 2013; Panganiban et al., 2013). In diabetes-induced atherosclerosis, high glucose-induced senescence, barrier disruption promotion in vascular endothelial cells, and maladaptive UPR constitute a pathway modulating the expression of senescence markers in diabetic conditions via ATF6 α and sXBP1 (Fatima et al., 2022). The DNA damage-inducible transcript 3 (DDIT3) protein level was significantly augmented in aged mouse hearts. Proprotein convertase subtilisin/kexin six is a proteolytic enzyme critical for maintaining cardiac function and vascular homeostasis. Its deficiency promotes cardiomyocyte senescence, which is related to DDIT3-mediated ER stress (Zhan et al., 2022). It can be inferred that ER stress and UPR can be either the reason or the result of senescence, considering that proteotoxic stress resulting from oxidized misfolded proteins or SASP protein overload is induced during senescence. Senescent cells may need optimal UPR activity for long-term survival (Abbadie and Pluquet, 2020). Pharmacological compounds inhibiting UPR subpathways may serve as senolytics for the cardiovascular system, although this remains to be verified.

Autophagy activation first happens in response to the stress stimulus. Therefore, it generally functions as an anti-senescence program. Enhancing autophagy in aged cardiomyocytes improves senescence (Li W. W. et al., 2021). Defective vascular smooth muscle cell (VSMC) autophagy boosts the development of stress-induced premature senescence and atherogenesis (Grootaert et al., 2018). Senescence is promoted in VSMCs. However, autophagy activation can suppress senescence in VSMCs (Li et al., 2014). Blocking autophagic flux and activating mTOR are involved in the mechanism of accelerating vascular senescence by the Yes-associated protein, which has been widely implicated in vascular pathophysiology processes (Pan et al., 2021). Similarly, augmented levels of ROS upon autophagy inhibition somewhat facilitate cellular senescence (Tai et al., 2017). However, another study showed that autophagy inhibition delayed but did not prevent senescence (Young et al., 2009). Autophagy produces a high flux of recycled amino acids and other metabolites, supporting massive synthesis of SASP factors and facilitating senescence (Kang and Elledge, 2016). This contradiction may be explained by the fact that autophagy may modulate some targets acting in an opposing manner to regulate cellular senescence. Autophagy inhibition can lead to diverse consequences depending on its timing, duration, or type.

Autophagy might crosstalk with ER stress and UPR to modulate senescence. Senescent microglia have lower autophagy and higher ER stress levels, possibly facilitating the progression of serious Alzheimer’s disease pathology (Angelova and Brown, 2018). Another study showed that UPR promotes autophagy and relieves acid-induced premature senescence in the nuclei of pulposus cells (Zhu et al., 2022). However, this connection is uncertain in CVDs and in the aging process.

### 3.4 ER stress, autophagy, and their overlap in mitochondria

Mitochondria are central organelles in the cell responsible for producing cellular energy. This role is especially crucial in the heart, where mitochondria take up around 30% of the overall cell volume and generate 6 kg of ATP every day for sustaining cardiac mechanical function (Torp et al., 2023). Mitochondria are also the chief source of ROS that modulate physiological processes and control cell death (Panel et al., 2018). The effectiveness of mitochondrial function decreases as the cells and organisms age. The reduction in mitochondrial function may greatly facilitate age-dependent changes in the cardiovascular system. For example, both human and animal models exhibited increased mitochondrial damage in atherosclerotic aortas (Ballinger et al., 2002). Mitochondrial dysfunction underlies atrial cardiomyocyte damage and brings about atrial fibrillation (Wiersma et al., 2019). Age-dependent mitochondrial DNA damage is a critical factor underpinning cardiac arrhythmia pathophysiology (Baris et al., 2015).

Both the ER and mitochondria are subjected to great metabolic demands (Green et al., 2014; Wang and Kaufman, 2016). Proteostasis and metabolic control are closely connected, and this interrelation is essential for maintaining healthy cardiac function (Murley and Nunnari, 2016). An imbalance in ER and mitochondrial interactions may be a prelude to cardiac aging and disease (Li et al., 2019). For example, although initial ER stress is an adaptive reaction for restoring cell functions, serious ER stress brings about electron transport chain injuries in adult heart mitochondria, contributing to mitochondrial dysfunction in aged hearts (Chen et al., 2020). Attenuating ER stress enhances mitochondrial functions in the aged hearts (Thompson et al., 2020).

Functional interaction between ER and mitochondria occurs through discrete ER-mitochondria contacts known as mitochondria-associated membranes (MAMs). Mitochondria in those domains are spatially and functionally organized in close contact with the ER, acting as a signaling hub that regulates cellular physiology (Carreras-Sureda et al., 2017). Commonly researched proteins involved in MAM formation include mitofusin-2 (Mfn2), which is a key component of mitochondrial fusion and fission (Chen et al., 2003). Cardiac-specific Mfn2 ablation decreased ER–mitochondrial tethering by 30%, decreasing mitochondrial Ca^2+^ uptake and hampering the reaction to physiological stress (Chen et al., 2012). Mfn2-deficient cells exhibit hyperactivated UPR signaling and defective autophagy and apoptosis, indicating the function of MAM integrity in UPR signaling (Muñoz et al., 2013). Another protein in the heart involved in the interaction between the ER and mitochondria is ER Ca^2+^ sensor stromal interaction molecule 1 (STIM1). It maintains ER Ca^2+^ levels in normal cardiac homeostasis. Mice with cardiomyocyte-specific STIM1 knockout showed pronounced ER dilatation, smaller mitochondria, and dysfunctional mitochondrial networks. They also developed cardiac fibrosis and dilated cardiomyopathy (Collins et al., 2014), potentially accelerating heart aging.

Mitochondrial quality control can also be achieved through mitophagy. Mitophagy protects cells from loss of mitochondrial function during aging. In the cardiovascular system, mitophagy induction in aged mice improves overall mitochondrial function and prevents arterial wall stiffness (LaRocca et al., 2014). Young mice with Parkin knockout have a normal cardiac phenotype, although abnormal mitochondria accumulate in cardiomyocytes with age (Kubli et al., 2013a). Inhibiting the PINK1/Parkin mitophagy pathway due to insufficient Drp1 induces myocardial apoptosis in the aging heart (Wei et al., 2021). This evidence revealed that the PINK1/Parkin pathway may mediate mitophagy in aging hearts.

Decreased mitophagy is also observed in aged hearts (Hoshino et al., 2013), while ER stress contributes to decreased mitophagy (Wang et al., 2017). Interestingly, MAMs have also been implicated in the control of mitophagy and mitochondrial integrity (Gelmetti et al., 2017). Mitophagy can be initiated in MAMs where PINK1 and Beclin-1 relocalize to promote ER–mitochondria tethering and autophagosome formation (Gelmetti et al., 2017). The mitophagy regulator FUNDC1 is also vital for MAM regulation in CVDs. FUNDC1 can be enriched in MAMs. Therefore, the formation of MAMs regulates cellular Ca^2+^ homeostasis and mitochondrial dynamics to prevent heart dysfunction (Li G. et al., 2021).

### 3.5 ER stress, autophagy, and their overlap in oxidative stress

Basal levels of ROS are essential for maintaining numerous cellular functions, including signal transduction pathway, gene expression, defense against invading microorganisms, growth promotion, and death (Finkel, 2011). Excessive ROS levels harm cellular macromolecules, eventually resulting in necrosis and apoptotic cell death. Oxidative stress occurs when the equilibrium of oxidant and antioxidant balance is disrupted and tends toward an oxidative state, where ROS are the main cause of oxidative stress. Oxidative stress is implicated in numerous pathological circumstances, neurodegenerative diseases, CVDs, diabetes, and cancers, most of which are aging-related and accelerate the aging rate (Wu et al., 2024). Excessive oxidative stress leads to endothelial dysfunction, enabling the permeation, trapping, and physicochemical modification of circulating lipoprotein particles in the subendothelial space (Gimbrone and García-Cardeña, 2016). ROS such as O^2−^ are able to react with nitric oxide (NO) to deplete NO bioavailability, triggering vasoconstriction in rats and leading to hypertension (Carlisle et al., 2016). Overexpression of mitochondria-targeted catalase protects the aged hearts (Bhuiyan and Fukunaga, 2009).

The interplay of ER and oxidative stress has been demonstrated in numerous pathological and physiological conditions (Zhang and Kaufman, 2008). Disulfide bond formation occurring in the ER is triggered by PDI and ER oxidase 1α (ERO1) (Tu and Weissman, 2004). PDI obtains electrons from protein-folding substrates and oxidizes the thiol group of cysteine residues, leading to disulfide bond formation. ERO1 mediates electron transfer from PDI to oxygen, resulting in ROS byproducts. To control oxidative stress, UPR pathways are activated by the adaptive mechanism to maintain cellular functions. In the heart, ATF6 serves as a unique bridge for linking ER stress to oxidative stress signaling pathways in cardiovascular stress conditions. In addition to its impact on ER protein folding, ATF6 can have an extensive influence on antioxidant protein expression even outside the ER (Jin et al., 2017). However, severe ER stress promotes ROS production and aggravates oxidative stress (Malhotra and Kaufman, 2007). In the heart, ER stress can diminish complex I activities and augment ROS generation (Chen et al., 2017; Chen Q. et al., 2019). On the other hand, disturbed redox homeostasis in the ER drives ER stress, causing ROS accumulation in the ER and mitochondria (Lei et al., 2017; Mennerich et al., 2019). Therefore, inhibiting ER and oxidative stresses can be a method of delaying heart aging. For example, swimming exercise inhibited oxidative and ER stresses in the hearts of aged mice by increasing cGMP-protein kinase G signaling (Chang et al., 2020).

The interplay between ROS and autophagy is crucial for cellular homeostasis, which is particularly critical in CVDs. Indeed, a physiological ROS level is needed for autophagy induction under conditions of nutrient deprivation and ischemia in cardiomyocytes. Nox4 is quickly activated in the ER in response to glucose deprivation in cardiomyocytes. Nox4-derived ROS boosts autophagy by driving the PERK/ATF4 pathway (Sciarretta et al., 2013). However, excessive oxidative stress can cause impairment in cardiac autophagy. For example, Nox2-derived superoxide activation mediates the impairment of autophagic flux subjected to lipid overload, and superoxide inhibition rescues autophagic flux in cardiomyocytes (Jaishy et al., 2015).

Autophagy is vital for reducing oxidative stress. Endothelial cell treatment with an autophagy inducer reduces oxidative stress (LaRocca et al., 2012). A prior study showed that endogenous CMA activator humanin protected cells from oxidative stress in aging-related CVDs (Cai et al., 2021). Notably, mitochondria are major producers of ROS, and appropriate removal of dysfunctional mitochondria is crucial as mitochondrial damage is linked to numerous cardiovascular pathologies (Lavandero et al., 2015). Through selective sequestration and degradation of dysfunctional mitochondria prior to those mitochondria causing damage or triggering cell death, mitophagy serves as a defense mechanism against ROS generation via aberrant mitochondria (Diao and Gustafsson Å, 2022). Given the importance of mitophagy, further studies are necessary to explore the role of mitophagy in ROS removal in the context of cardiac aging and CVD to identify additional targets for intervention.

The decline in the Nrf2/EpRE signaling system is vital for aging and aging-related cardiovascular oxidative stress (Gounder et al., 2012; Volonte et al., 2013; Li et al., 2023). Autophagy is crucial in ROS scavenging through activation of Nrf2 by p62 (Komatsu et al., 2010). Oxidative stress induces ER stress, amplifying ROS production and causing autophagy upregulation, whereas Nrf2 is tightly linked to ER stress signals (Cominacini et al., 2015; Wang et al., 2020). Nrf2 may be the hub to coordinate UPR and autophagy during oxidative stress, but a direct connection among them needs to be further investigated, particularly in aging-related CVDs.

### 3.6 ER stress, autophagy, and their overlap in nutrient-sensing pathways

Cardiac aging is related to impairment in some nutrient and metabolic pathways, possibly directly or indirectly impacting functional and structural deterioration of the heart (Daneshgar et al., 2021). The mTOR pathway is a critical signaling pathway. It forms two distinct complexes, mTORC1 and mTORC2, where mTORC1 is an important signaling hub coordinating nutrient status and cell growth (Kim and Guan, 2019). The involvement of mTOR during aging was initially demonstrated within a *Caenorhabditis elegans* model system, showing decreased expression of mTOR homologs or Raptor-extended life span (Jia et al., 2004). Activation of mTOR upregulates the expression of genes involved in growth and metabolism, downregulates genes related to cellular stress adaptation, and suppresses catabolic processes, such as autophagy (Saxton and Sabatini, 2017; Battaglioni et al., 2022). In the heart, chronic mTOR activation may increase cardiac aging. Activating autophagy via mTORC1 inhibition provides potential beneficial effects during aging. Aged mice undergoing 3-month mTOR inhibitor rapamycin treatment showed alleviated cardiac aging pathologies, such as cardiac fibrosis and heart inflammation, and improved cardiovascular function (Flynn et al., 2013). In contrast, chronic Akt1 activation led to activated mTORC1, worsening aging-induced cardiac hypertrophy and myocardial contractile dysfunction via inhibition of autophagy (Hua et al., 2011). Mice with systemic GSK-3α deletion showed cardiac hypertrophy, dysfunction, and sarcomere abnormalities during aging due to deregulated mTORC1 activation and autophagy inhibition (Zhou et al., 2013). However, current research has demonstrated that rapamycin prolongs the lifetime but failed to show that it prevents cardiovascular aging (Neff et al., 2013). This inconsistency in the impact of mTORC1 inhibition on the aging heart is possibly due to diverse cell forms and sources of mTORC1 inhibitors.

ER stress and the mTOR signaling network function in a coordinating manner to regulate various cellular processes, cell development, and survival. ER stress induces mTORC1 activation within the NRK-52E cells, leading to activation of the IRE1-JNK pathway and apoptosis. Inhibition of mTORC1 weakens ER stress-induced apoptosis by selectively suppressing IRE1-JNK signaling and enhancing cellular survival in ER stress (Kato et al., 2012). Constitutive mTORC1 activation by loss of TSC stimulates JNK, contributing to ER stress-induced apoptosis (Ozcan et al., 2008; Bachar et al., 2009). mTORC1 also functions as a feedback loop to regulate ER stress signaling, as observed in intestinal epithelial cells incubated with tunicamycin. This exposure resulted in ER stress-induced caspase-3-dependent apoptotic cell death, which was enhanced by rapamycin (Ji et al., 2018). To date, there is insufficient research devoted to defining ER stress and mTOR signaling networks in cardiac aging and disease. The mTOR-autophagy-ER stress axis may exist in aging and pathological conditions in the cardiovascular system. In a murine model of cardiac hypertrophy, inhibiting sodium-glucose co-transporter two can reduce left ventricular fibrosis, which is related to reduced cardiac insulin level, augmented AMPK signaling inhibition of cardiac mTOR activation, and a reduction in downstream ER stress, UPR, and apoptosis (Moellmann et al., 2022). In addition, rapamycin decreased cardiomyocyte apoptosis and boosted cardiomyocyte autophagy. This protective effect was modulated by adjusting crosstalk between the mTOR and ER stress pathways in chronic heart failure (Gao et al., 2020). In blood vessels, inactivation of CCAAT/enhancer-binding protein beta attenuates macrophage foam cell formation in atherogenesis by diminishing inflammation, ER stress, and apoptosis, boosting autophagy, and inactivating mTOR (Zahid et al., 2020). In addition, mTOR-autophagy dysfunction activated ER stress, thus inducing lipid metabolism, inflammation, and fibrosis (Wang et al., 2022).

### 3.7 ER stress, autophagy, and their overlap in DNA damage

DNA damage accumulation is a strong candidate for the primary cause of aging by inducing cell death, senescence, and tissue dysfunction (Schumacher et al., 2021; Zhao et al., 2023). Therefore, cells have developed an intricate mechanism for confronting and repairing DNA lesions, which is known as DNA damage repair (DDR). It involves one coordinated network that can be triggered by either single-strand breaks (SSBs) or double-strand breaks (DSBs) in the DNA. Reaction to DSBs is mediated by three kinases that are members of the PI3K-related kinase family. They include ATM, ATR, and DNA-PKcs (Blackford and Jackson, 2017), which coordinate the phosphorylation of various proteins, regulating a broad spectrum of cellular processes involved in DNA repair and replication, cell-cycle control, and apoptosis. SSB lesions produced at DSB sites or collapsed replication forks can cause PARP1 and ATR activation (Ciccia and Elledge, 2010). More clinical and preclinical evidence demonstrates the presence of DNA damage and activation of DDR signaling in the growth and/or progression of CVDs, though these processes remain largely underexplored. For example, DDR is activated within postmitotic cells like cardiomyocytes (Botto et al., 2001). Human atherosclerotic plaques show more DSBs and ATM activation than normal tissue (Mahmoudi et al., 2008). An age-associated increase in mitochondrial ROS causes mitochondrial DNA (mtDNA) mutations. Plasma levels of mtDNA fragments and mtDNA damage remain high in CVD patients (Nie et al., 2020), and mtDNA mutations may be highly associated with heart aging (Quan et al., 2020).

Although ER stress, UPR, and DDR are adaptive mechanisms that occur in the ER and nucleus, there is emerging evidence showing that signaling crosstalk exists between UPR stress sensors and DDR. For example, sXBP1 directly controls transcription of various DDR genes and γH2AX levels (Argemí et al., 2017). sXBP1 silencing leads to an increase in γH2AX foci formation and a decrease in the expression of MRN complex proteins and ATM phosphorylation (Lyu et al., 2019). Selective activation of IRE1α under genotoxic stress regulates the repair program and maintains cell survival. Genotoxic agents are involved in RIDD activity without XBP1 mRNA splicing (Dufey et al., 2020). This IRE1α is involved in mRNA degradation and plays a role in DDR, influencing DNA repair, cell-cycle arrest, and cell death. PERK-p-eIF2α-ATF4 signaling activation via SSBs has been shown to support cell survival under nutrient-restricted conditions (Clementi et al., 2020). The molecular connection between UPR and DDR, which maintains both genome integrity and proteostasis in CVDs, is not fully characterized. However, the evidence above suggests a potential coordination between the two pathways that may be relevant to aging and CVDs. Future experimental evidence is needed to explore this idea.

Autophagy is also involved in the reaction to genotoxic stress. Autophagy upregulation has been observed in response to cellular DNA damage (Juretschke and Beli, 2021), while DNA damage accumulation has been shown in autophagy-deficient cells and mice (Schneider et al., 2014). Autophagy modulates the DNA damage response via p62 degradation (Wang et al., 2016). It also directly targets DNA-associated proteins (Chen S. et al., 2015) and DNA resection proteins (Robert et al., 2011). In addition, nucleophagy, which is the process of selective degradation of nuclear components, has been observed in yeast and mammals (Mijaljica et al., 2012; Li and Nakatogawa, 2022). Autophagy upregulation in DNA damage in the heart is mediated via ATM, PARP1, and cGAS-STING signaling. ATM is vital for myocyte apoptosis and cardiac remodeling following myocardial infarction. ATM deficiency results in autophagic impairment in myocardial infarction (Thrasher et al., 2018). PARP1 is involved in autophagy induced by DNA damage (Muñoz-Gámez et al., 2009). Cardiomyocytes in patients suffering from persistent atrial fibrillation demonstrate significant DNA damage that is related to PARP1 activity (Zhang et al., 2019). Inhibiting PARP1 to activate autophagy might serve as an effective strategy to preserve cardiomyocyte function. The cGAS-STING signaling axis senses extranuclear chromatin due to genotoxic stress and DNA released from the mitochondria (Hopfner and Hornung, 2020). The cGAS-STING signaling pathway has been implicated in cellular senescence (Lan et al., 2019) and cardiac hypertrophy (Zhang Y. et al., 2020) through direct and indirect recognition of aberrant self-DNA. Autophagy has a conserved function in the cGAS-STING pathway (Gui et al., 2019). STING-mediated autophagy was discovered to be essential for removing cytosolic DNA following DNA damage. Autophagy/mitophagy inhibits STING signaling and its excessive responses (Oduro et al., 2022). This negative feedback controlling STING signaling ameliorates diabetic cardiomyopathy (Lu Q. B. et al., 2022).

Unc-51-like kinase (ULK) is essential for initiating autophagy in response to DNA damage. ULK1 is a transcriptional p53 target enhancing PARP1 activity, causing sustained autophagy and cell death (Gao et al., 2011; Joshi et al., 2016). ULK also mediates the phosphorylation of its interaction partner SEC16A and regulates the assembly of ER exit sites and ER-to-Golgi trafficking of specific cargo. ULK deficiency results in ER-to-Golgi trafficking defects and activates the UPR pathway (Joo et al., 2016). ULK1 may serve as the nexus that links autophagy, UPR, and DDR. However, direct data demonstrating this mechanism are still needed, particularly regarding its implications in CVDs.

## 4 ER stress, UPR, and autophagy in aging-related CVDs

### 4.1 Atherosclerosis

Advancing age remains the primary risk factor for atherosclerosis (Tyrrell and Goldstein, 2021). Multiple aging-associated risk factors, including oxidative stress, inflammation, hyperhomocysteinemia, and free cholesterol accumulation in macrophages, are involved in atherosclerosis (Fu et al., 2018; Forman and Zhang, 2021; Tyrrell and Goldstein, 2021). The potential mechanism for these pro-aging risk factors suggests that they couple with activators in ER stress and maladaptive UPR, further facilitating atherosclerosis etiology (Yang et al., 2018; Jiang et al., 2021). ER stress and its response play a role in atherogenesis in coronary artery lesions, as demonstrated by augmented levels of ER stress markers like BiP and CHOP (Myoishi et al., 2007). Mice with CHOP-deficiency crossed with *Apoe*- or *Ldlr*-knockout mice experienced decreased necrosis in atherosclerotic plaques and reduced atherosclerotic lesion areas (Thorp et al., 2009). Type 2 diabetes mellitus (T2DM) is hallmarked by accelerated atherosclerosis (La Sala et al., 2019), and nearly half of all individuals with T2DM are older adults. High glucose levels induce more pronounced responses related to maladaptive UPR, senescence, and vascular endothelial cell barrier disruption. Direct evidence for the mechanism of ER stress in aged animal models requires further study.

Autophagy is vital for regulating atherosclerosis. CMA function becomes impaired in atherosclerosis progression, leading to NLRP3 inflammasome activation and IL-1β secretion and promoting vascular inflammation and atherosclerosis progression (Qiao et al., 2021). Pharmacological activation of CMA provides a novel therapeutic method for atherosclerosis. Aging-related decreases in vascular mitochondrial functions and impaired mitophagy are vital for chronic hyperlipidemia and atherogenesis (Tyrrell and Goldstein, 2021). Autophagy also helps to maintain plaque cells, protecting them against oxidative stress, which can accelerate aging and is a hallmark of advanced atherosclerotic lesions (Yang et al., 2023). Autophagy stimulates the survival of VSMCs, whereas reduced autophagy promotes age-related changes in the vasculature. Defective VSMC autophagy accelerates the development of stress-induced premature senescence and atherogenesis (Grootaert et al., 2018).

Macrophage ATG5 deficiency in fat-fed Ldlr(−/−) mice increases apoptosis and oxidative stress in advanced lesional macrophages, promotes plaque necrosis, and exacerbates lesioned efferocytosis (Liao et al., 2012). Elevated cholesterol levels in mouse macrophages stimulate ER stress-induced apoptosis and promote autophagy using rapamycin-increased cholesterol efflux in oxLDL-loaded macrophages (Zahid et al., 2020). Therefore, autophagy may be a protective stress reaction to ER stress, apoptosis, and inflammation in regulating macrophage foam cell formation.

### 4.2 Cardiac hypertrophy and heart failure

Aging is a major risk factor for heart failure (Florio et al., 2020). Adaptive UPR is able to maintain homeostasis of the heart (Steiger et al., 2018). Aged mouse hearts showed much lower EIF2A phosphorylation levels and lower BiP expression levels while expressing the proapoptotic factor CHOP at high levels. ER stress responses may be the mechanisms involved in the protective effect of folic acid against cardiac aging (Ye et al., 2021). However, ER stress and maladaptive UPR may facilitate cardiac hypertrophy and heart failure via activation of PERK and CHOP signaling. ER and UPR component levels, such as XBP1s, CHOP, and ATF4, are elevated in patients suffering from heart failure (Yao et al., 2017; Schiattarella et al., 2021). Aging leads to increased ER stress, which contributes to mitochondrial dysfunction, while mitochondrial ROS generation also induces ER stress. Targeting ER stress and calpain activation may reverse age-dependent mitochondrial damage in the heart (Thompson et al., 2020).

Autophagy can increase protein degradation, while autophagy inhibition in cardiomyocytes promotes heart failure progression due to proteotoxicity (Delbridge et al., 2017). Mice with Parkin deficiency may exhibit normal cardiac functions while young (Kubli et al., 2013b) and accumulate abnormal mitochondria during aging (Kubli et al., 2013a). Aging-related cardiac remodeling is also related to autophagy dysfunction (Linton et al., 2015; Shirakabe et al., 2016). Autophagy activation via caloric restriction (CR) delays the development of aging-related cardiac disorders (Makino and Maeda, 2021). However, cardiomyocyte-specific autophagy activation through αMHC-Beclin-1 overexpression promotes hypertrophic remodeling and systolic dysfunction (Zhu et al., 2007). Autophagic cell death is also implicated in the pathological process of heart failure (Ding et al., 2020).

UPR signaling and autophagy are interrelated. The PERK/ATF4 signaling activation and autophagy defects promote cardiomyocyte hypertrophy and cardiac fibroblast activation (Li et al., 2018b). However, ER stress activation through PERK-ATF4 can cause lethal autophagy-mediated cardiac atrophy (Vanhoutte et al., 2021).

### 4.3 Hypertension

The National Health and Nutrition Examination Survey indicated that 70% of people older than 60 have hypertension compared to only 32% of those aged 40–59 years (Mozaffarian et al., 2015).

ER stress is vital for vascular cell phenotype switching, dedifferentiation, calcification, and apoptosis and facilitates endothelial dysfunction and vascular remodeling in hypertension (Shanahan and Furmanik, 2017; Griendling et al., 2021). ER stress inhibition blunts hypertension growth among spontaneously hypertensive rats (Spitler et al., 2013; Naiel et al., 2019) and protects against hypertension-induced vascular dysfunction (Carlisle et al., 2016). Direct data for different effects of ER stress on aged mice or older patients and its potential regulation mechanisms remain limited.

Autophagy is involved in numerous pathological processes related to hypertension and target organ injuries. Aging is associated with downregulation of the Klotho protein, which favors autophagy-suppressed aging (Fernández et al., 2018). Klotho deficiency-triggered arterial stiffening is due to decreased elastin and increased collagen-1 levels and involves compensatory autophagy induction (Chen K. et al., 2015). Autophagy reactivation may remove dysfunctional mitochondria and limit damage to endothelial and cerebral cells in response to high salt level treatment, thereby reducing hypertension-related stroke occurrence (Forte et al., 2020).

The relationship between ER stress and autophagy during hypertension remains poorly defined, and future studies are needed to explore their regulation in aging-associated blood vessel changes and hypertension.

## 5 Targeting aging and CVDs by modulating ER stress and autophagy

Due to the pivotal function of ER stress and autophagy during aging and CVD pathogenesis, methods to target proteins and signaling components involved in these cellular processes are attractive therapeutic avenues for disease interventions.

### 5.1 Pharmacological interventions

#### 5.1.1 Metformin

Metformin is a well-established and inexpensive principal anti-diabetic drug (Shin et al., 2020). Its use can lead to a 21% reduction in mortality in myocardial infarction and a 16% reduction in mortality in heart failure (Han et al., 2019). Metformin stimulates the AMPK/PPARδ pathway to inhibit ER stress, enhances cardiac injuries, and endothelial dysfunctions and maintain vascular health (Cheang et al., 2014; Ge et al., 2019). In particular, it attenuates ER stress and guards against vascular damage in hypertension (Chen C. et al., 2019). It also inhibits mTOR signaling to promote autophagy and prevent atherosclerosis development (You et al., 2020).

#### 5.1.2 Sirtuin activators

Sirtuins are a family of nicotine adenine dinucleotide (NAD+)-dependent histone deacetylases, which are referred to as “the fountain of youth” due to their critical function in longevity and aging anomalies (Kanfi et al., 2012; Verdin, 2015). SIRT1 is a master regulator of cellular stress. It guards cardiomyocytes from ER stress-induced cell death by modulating the PERK/eIF2*α* pathway of UPR via deacetylation of eIF2*α* on lysine K143 (Prola et al., 2017). Cardioprotective SIRT1 influences are also caused by its capability to affect autophagy by directly deacetylating autophagy machinery, such as ATG5, ATG7, and LC3 (Lee et al., 2008). SIRT1 may play a cardioprotective role in enhancing autophagy induced by ER stress by activating the eEF2K/eEF2 pathway (Pires Da Silva et al., 2020).

#### 5.1.3 Statins

Statins are effective cardioprotective drugs because they can inhibit HMG-CoA reductase. In addition, statins have beneficial pleiotropy anti-thrombotic, anti-oxidative, and anti-inflammatory effects (Oesterle et al., 2017). The beneficial impact of statin therapy on cardiac remodeling due to pressure overload and heart failure in rats is associated with ER stress reduction (Song et al., 2011). Rosuvastatin exerts a cardioprotective effect by enhancing the autophagy pathway by increasing Beclin-1 expression and MAPK activation and inhibiting mTOR signaling (Song et al., 2018).

#### 5.1.4 mTOR inhibition

A previous study discovered that ginkgo biloba leaf extract alleviates atherosclerosis in streptozotocin-induced diabetic ApoE^−/−^ mice by restraining ER stress via autophagy restoration using the mTOR signaling pathway (Tian et al., 2019). Therefore, this pathway is a possible target for modulating ER stress in CVDs.

### 5.2 CR and lifestyle modification

#### 5.2.1 Caloric restriction

Hearts in patients undergoing caloric restriction (CR) exhibit mTOR suppression and activated autophagy. CR-elicited autophagy has a beneficial effect on cardiac aging (Makino and Maeda, 2021). Patients with obesity undergoing CR lose weight and alleviate ER stress, as demonstrated by a decrease in phosphorylated ATF6, CHOP, and JNK levels (López-Domènech et al., 2019). Low-level inhibition of fat consumption in rats can restore AMPK activities in adipose tissues, liver, and peripheral blood mononuclear cells by inhibiting ER stress and UPR modulators CHOP, EIF2A, PERK, and XBP1 (Vega-Martín et al., 2020).

#### 5.2.2 Physical exercise

Swimming has been shown to diminish ER stress and ROS production in mice and attenuate aging-related CVDs and cardiac dysfunctions by activating cGMP-protein kinase G signaling (Chang et al., 2020). Exercise also diminishes atherosclerosis plaques and boosts endothelial function by reducing ER stress, which is closely related to endothelial dysfunctions in diabetes and atherosclerosis (Cheang et al., 2017; Hong et al., 2018). Furthermore, analyses of human skeletal muscle biopsy samples revealed that exercise is related to the adaptive UPR in muscle remodeling and is impaired during aging (Hart et al., 2019).

Exercise augments acute autophagic activities in skeletal muscle as well as several other tissues, including the heart (Escobar et al., 2019). This mechanism involves increased Sirtuin 3 and PINK1/Parkin levels (Zhao et al., 2018). In addition, the combination of long-term exercise together with CR may be more effective in activating autophagy and thus preventing heart failure than either therapy used alone (Moradi et al., 2019).

## 6 Conclusions and perspectives

Aging is a complex and multifaceted process that results in widespread functional decline affecting every organ and tissue. Determining the mechanisms by which aging enhances CVDs is important to identify new treatments that decrease the burden of aging-related CVDs.

Many biological processes change as a result of aging, contributing to an increased risk of CVDs. ER stress and autophagy are two typical cellular stress response pathways that can function synergistically during aging and aging-associated diseases. As individuals age, many components of the ER stress and autophagy pathways diminish and become less effective, thereby decreasing their reaction potential to various stressors and leading to or exacerbating existing diseases. ER stress response and autophagy pathways and their age-related modifications are also implicated in various other aging-related diseases in a variety of organs, including Alzheimer’s disease, Parkinson’s disease, type 2 diabetes mellitus, and cancer involving disturbed proteostasis. ER stress and impaired autophagy flux are involved in neuronal degeneration and brain injury. A neuroprotective agent, docosahexanoic acid, has unique functions by reducing ER and oxidative stress and modulating autophagy (Yin et al., 2017). In Alzheimer’s disease, ER stress response and impaired autophagy take part in cellular aging and neuroinflammation (Reddy and Oliver, 2019; Uddin et al., 2021). Therefore, coupling UPR, autophagy, and inflammation can facilitate novel therapeutic strategies to mitigate cellular stress and inflammation, which are involved in chronic obstructive pulmonary disease and neurodegenerative disorders (Chipurupalli et al., 2021). Harnessing ER stress response and autophagy is of broader significance for the prevention and treatment of more age-related diseases than CVDs.

Both ER stress and autophagy are associated with one or more cell death processes (Liu et al., 2023; An et al., 2024). Whether they are connected in aging and CVDs by modulating cell death processes is a topic that warrants further study.

Although our awareness of the roles of these aging processes and their impact on CVD progression has recently improved, detailed molecular mechanisms and cellular pathways remain under investigation, with critical issues still unresolved. Therefore, further exploration of the precise mechanisms governing the interplay of ER stress and autophagy and their overlap with aging risk would help elucidate the pathways involved in cellular homeostasis maintenance in the cardiovascular system. Such research could aid with therapeutic development targeted at maintaining a normal ER stress reaction and autophagy for later life, potentially delaying or preventing aging-related CVDs. This represents an unexplored avenue in CVD prevention.
